# Supplementary material for: Conditional chemoconnectomics (cCCTomics) as a strategy for efficient and conditional targeting of chemical transmission
Source: eLife. 2024 Apr 30;12:RP91927. doi: 10.7554/eLife.91927 (PMC11060718; doi:10.7554/eLife.91927)
Supplement: Figure 3—source data 3. [file elife-91927-fig3-data3.zip › Figure 3 source data3 part 2.zip!/Figure 3-Source data 3-part2/1053f2-1-CRISPResso_Report_y3hlioj1.zip!/CRISPResso_on_1053f2_4/1c.Alignment_barplot.pdf]

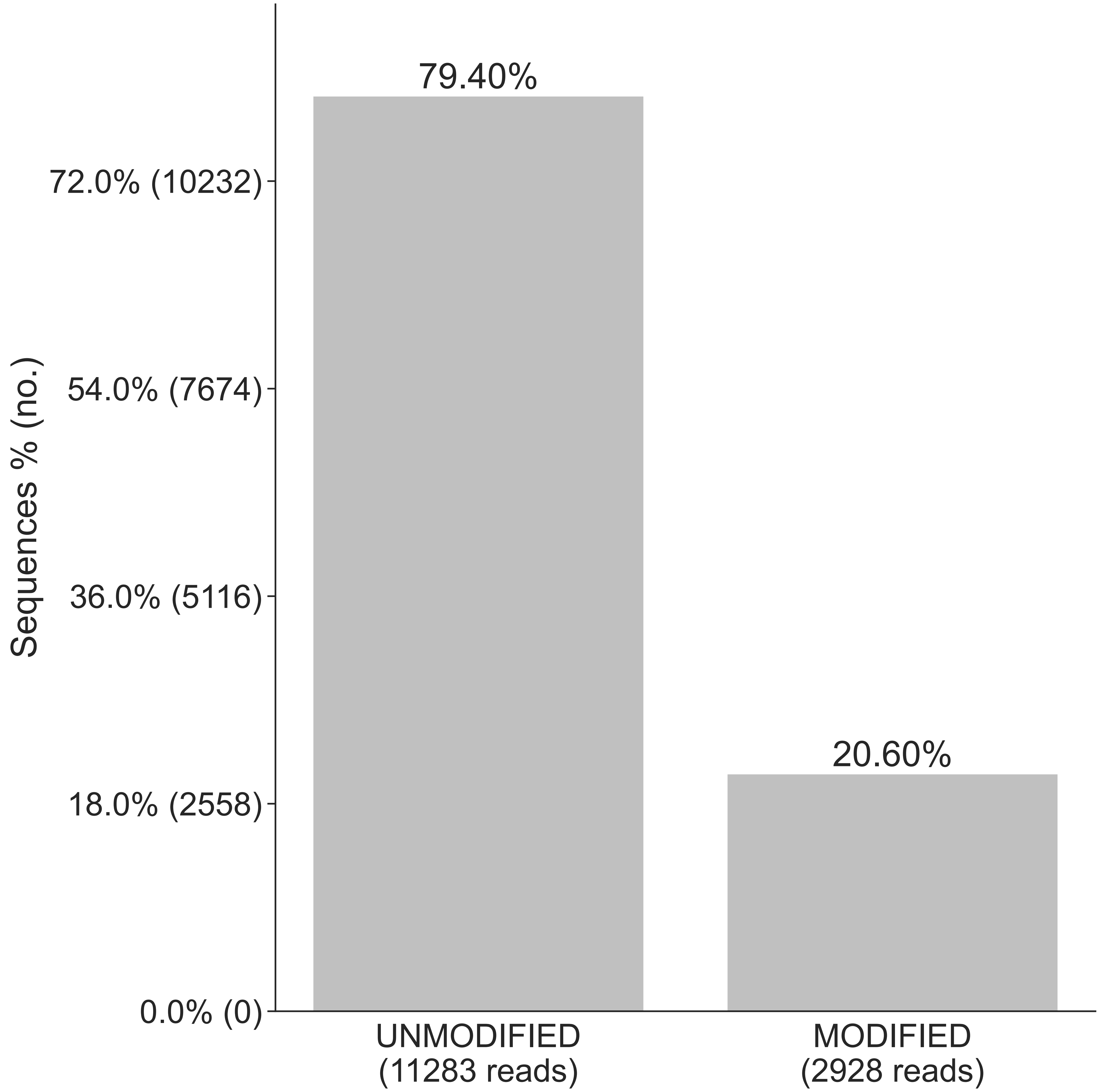

79.40%
20.60%
Sequences % (no.)
72.0% (10232)
54.0% (7674)
36.0% (5116)
18.0% (2558)
0.0% (0)
UNMODIFIED
(11283 reads)
MODIFIED
(2928 reads)
